# Reticulospinal and corticospinal responses in long-term strength-trained and untrained adults

**DOI:** 10.1007/s00421-025-05834-x

**Published:** 2025-06-10

**Authors:** Meghan Tanel, Dawson J. Kidgell, Sakari Vekki, Juha P. Ahtiainen, Gonzalo Gomez-Guerrero, Eeli J. Halonen, Stuart N. Baker, Simon Walker

**Affiliations:** 1 NeuroMuscular Research Center, Faculty of Sport and Health Sciences, University of Jyväskylä, PO Box 35 (VIV225), Jyväskylä, Finland; 2 Monash University Exercise Neuroplasticity Research Unit, School of Primary and Allied Health Care, Monash University, Melbourne, Australia; 3 Institute of Biosciences, Faculty of Medical Sciences, Newcastle University, Newcastle Upon Tyne, UK

**Keywords:** Neural function, StartReact, TMS, Startle, Force

## Abstract

Studies investigating neural adaptation to strength training have yet to determine the loci of previously observed improvements in motor pathway function. While transcranial magnetic stimulation (TMS) studies have typically focused on the corticospinal tract, recent evidence from primates suggests that the subcortical reticulospinal tract may be the primary candidate for adaptation. Methods: Using a cross-sectional comparison, long-term strength-trained athletes (*n* = 15, 11M/4F, 32 ± 6 y) and untrained adults (*n* = 18, 11M/7F, 32 ± 4 y) underwent a series of neurophysiological tests designed to target corticospinal and reticulospinal functioning based on biceps brachii muscle responses. The StartReact test was used to determine reaction time, rate of torque development, and muscle activity in response to visual (V), visual–auditory (VA, 80 dB), and visual–startle (VS, 120 dB) cues. Reaction times were used to calculate reticulospinal gain ([V–VS]/[V–VA]). Neuro-navigated monophasic TMS to the motor cortex was given during low-force level isometric voluntary contractions (10% of maximum) with both posterior–anterior (100–180% active motor threshold) and anterior–posterior currents (120% active motor threshold). Motor evoked potentials (MEPs) from stimuli given at 120% active motor threshold were compared to startle cue-conditioned responses and anterior–posterior current responses. Results: Untrained adults benefitted more from StartReact (reticulospinal gain, *p* = 0.003), whereas strength-trained athletes showed greater MEP size at higher stimulation intensities (180% active motor threshold, *p* = 0.048, 140–180% area-under-the-curve, *p* = 0.020) and shorter silent period (180% active motor threshold, *p* = 0.035). Conclusions: Evidence provided here suggests greater cortico-reticulospinal excitability/functioning in long-term strength-trained athletes.

## Introduction

Strength training is a widely recommended method to increase maximal strength. Both neural and muscular adaptations underpin functional gains (e.g., increased maximal force production) from strength training, although adaptations during the early weeks of a training program are considered to be predominantly of neural origin (Carroll et al. 2002; Moritani and DeVries 1979; Sale 1988). Augmented neural excitability has been observed cortically and subcortically as a result of short-term strength training (Kidgell et al. 2017; Siddique et al. 2020). However, it remains unclear which descending motor pathways are responsible for neural adaptations to strength training.

The corticospinal tract (CST) has been the primary candidate for mediating early adaptations to strength training. Corticospinal excitability is a major contributor to descending drive and spinal motor neuron pool activation, both of which influence force production (Del Balso and Cafarelli 2007; Duchateau et al. 2006). Neuromodulatory techniques such as transcranial magnetic stimulation (TMS), a form of non-invasive brain stimulation (Barker et al. 1985), have been used to evaluate changes in intracortical and CST function after a strength training intervention. When stimulating the primary motor cortex, peripheral responses to TMS known as motor evoked potentials (MEPs) are often used to quantify intracortical and CST functioning. Investigations into neural adaptations to strength training have predominantly conducted short-term interventions. Short-term strength training (3–5 weeks) of the upper-limbs has been shown to augment CST excitability (Kidgell et al. 2010; Mason et al. 2017; Pearce et al. 2013) and reduce corticospinal (Hendy and Kidgell 2013; Kidgell and Pearce 2010; Mason et al. 2017) and intracortical inhibition (Hendy and Kidgell 2013). However, several contrary studies have presented negligible changes (Lee et al. 2009; Leung et al. 2017) and even attenuation (Jensen et al. 2005) of intracortical and CST excitability following upper-limb short-term strength training despite improvements in strength of the trained muscle. Neural adaptations to long-term strength training, particularly in elite-level strength athletes, remain insufficiently investigated.

Equivocal findings from traditional TMS tests after short-term strength training could be influenced by variation in training and TMS testing protocols between studies. Alternatively, these findings may indicate that there are other neural mechanisms underpinning early functional gains. The reticulospinal tract (RST), a motor pathway projecting bilaterally from motor nuclei in the pontomedullary reticular formation to innervate alpha (α)-motor neurons of both proximal and distal upper-limb muscles (Baker 2011), could be an alternative descending tract mediating increases in muscle strength (Akalu et al. 2023; Atkinson et al. 2022). Currently, human-based evidence for reticulospinal adaptation to strength training is minimal, primarily due to the limited methods for stimulating the tract’s subcortical origin.

Startling auditory stimuli have been used to activate the reticular formation and elicit a startle response in the RST (Davis et al. 1982; Tapia et al. 2022; Valls-Solé et al. 1995). RST activation by loud acoustic stimulation is thought to involuntarily release a planned motor response (Carlsen et al. 2004). Practically, this mechanism has been applied in the StartReact test, where loud acoustic stimulation induces reduced voluntary reaction times to a visual cue compared to a quiet sound (Valls-Solé et al. 1995, 1999). Studies that have found enhanced StartReact in populations with corticospinal impairments (i.e., stroke and spinal cord injury) provide further evidence for RST influence as a compensatory mechanism (Baker and Perez 2017; Choudhury et al. 2019). Separately from StartReact, loud acoustic stimulation has also been paired with TMS during steady submaximal voluntary contraction (e.g., 10% maximal voluntary force), resulting in transient suppression of MEPs at an interval of ~ 50 ms (Fisher et al. 2021; Furubayashi et al. 2000). This may reflect an interaction between a cortical suppression and reticulospinal facilitation (Tapia et al. 2022); decreases in the net suppression may, therefore, serve as an additional non-invasive assessment of RST excitability (Germann et al. 2023). Finally, insight into subcortical systems may be obtained using TMS by inducing an anterior–posterior (AP) current. AP current stimulation evokes motor responses with greater contribution from later indirect (I)-waves compared to the posterior–anterior (PA) orientation, possibly reflecting more indirect pathways with subcortical synapses (Cirillo and Perez 2015; Federico and Perez 2017).

To date, the most compelling data to support RST adaptation to strength training are from non-human primates, which have found that a short-term strength training program induced MEP facilitation from motor cortical and reticular formation stimulation, but not direct corticospinal stimulation (Glover and Baker 2020), suggesting changes in both intracortical and reticulospinal neurons. Few studies in humans have been designed to investigate reticulospinal function using the available non-invasive tests. Notably, rock climbers had a greater rate of force development in response to the loud sound cue than untrained participants during a gripping task in the StartReact test, which indicates enhanced RST output (Colomer-Poveda et al. 2023). Our recent cross-sectional study examining cortical, corticospinal, and reticulospinal excitability of the flexor carpi radialis muscle in adults with 2 year experience showed enhanced intracortical and RST excitability compared to untrained adults (Akalu et al. 2024). While these findings are consistent with primate data (Glover and Baker 2020), further research involving long-term strength-trained individuals, with the goal of maximizing strength performance, and complementary neurophysiological assessments is needed to substantiate this hypothesis and to identify appropriate measures of underlying neurophysiology. Therefore, the aim of this study was to investigate whether strength training status affects corticospinal and reticulospinal excitability. A series of tests, including the StartReact and single-pulse TMS paradigms, were used to ascertain motor pathway excitability in long-term strength-trained compared to untrained adults.

## Methods

### Participants and experimental setup

A total of 33 healthy young adults participated in the study, 15 of which were long-term strength-trained and the remaining 18 were untrained. The trained group was comprised of competitive strength-trained athletes (weightlifters, powerlifters, and weighted pull-up athletes (*n* = 5), bodybuilders and fitness athletes (*n* = 6), gymnasts (*n* = 2), and an Olympic rower (*n* = 1)) and long-term strength-trained participants competitive in non-strength specific sports (*n* = 2). All strength-trained participants had to have at least 3 years of systematic strength training experience and, importantly, had won medals in their respective sports at national and/or international level. The age-matched untrained group was recreationally active, mostly participating in aerobic exercise, but did not engage in regular strength training. All participants were without neurological or musculoskeletal disorders, hearing difficulties or use of a hearing aid, or contraindications to TMS outlined by Rossi et al. (2021). Participants were classified as right-handed based on verbal confirmation to the question, “Which hand would you primarily throw a ball with?” Ethical approval was granted by the Ethics Committee of the University of Jyväskylä, Finland (1592/13.00.04.00/2022). Informed consent was obtained from all participants prior to measurements and the study was conducted in accordance with the declaration of Helsinki, apart from registration in a database.

This cross-sectional study included one laboratory visit. Body composition parameters were measured using multifrequency bioelectrical impedance analysis (InBody 770 Body Composition Analyzer, BioSpace Co Ltd, Seoul, South Korea). This test was used to obtain total skeletal muscle mass (SMM) to compare between groups, as well as right arm SMM to account for the contribution of muscle mass on maximum force production. Participants were seated for the remainder of the study protocol with the right hand in a supinated position, elbow flexed to 90°, and shoulder at 0°. The right arm was secured at the wrist to a strain gauge built into a custom isometric electromechanical dynamometer (University of Jyväskylä, Finland).

### Electromyography and force recordings

Bipolar surface Ag/AgCl electrodes (22 × 44 mm, Ambu BlueSensor N, Denmark) were placed on the belly of the right biceps brachii according to SENIAM guidelines (Hermens et al. 1999) following skin preparation. Inter-electrode distance was 20 mm, impedance was < 2 kΩ, and the ground electrode was positioned on the olecranon. Electromyography (EMG) signals were amplified (500 gain) and bandpass filtered at 16–1000 Hz (NeuroLog system NL844, Digitimer Ltd, Welwyn Garden City, UK) prior to being sampled at 5000 Hz. Force was measured at a sampling rate of 1000 Hz. Force and EMG signals were passed to a 16-bit A–D converter (Power1401-3, CED, Cambridge, UK) and recorded using Spike2 software (version 8.11b, CED, Cambridge, UK).

### Maximal voluntary contractions

Following several submaximal warm-up contractions, participants were instructed to perform unilateral isometric elbow flexion “as hard and as fast as possible”. Force feedback was provided on a monitor 1 m in front of the participant. Three contractions of approximately 3 s were performed with at least 30 s rest in between. Further trials were conducted if maximal force production of the third trial was at least 5% greater than the previous two trials. Contraction onset was automatically set at a threshold of 5 N above mean force 100 ms prior and visually confirmed. Maximal voluntary contraction (MVC) was determined as the trial with the greatest peak force production without countermovement (> 0.5 N; Maffiuletti et al. 2016). Force values for MVC (N) were converted to torque (Torque_max_, N·m) based on each individual’s lever arm distance measured from the humeroradial joint space to the center of the strain gauge at the wrist. Maximal rate of torque development (RTD_max_) was calculated as the slope of the force–time curve over the first 100 ms and then converted to torque (N·m·s^−1^) based on each individual’s lever arm distance.

### Motor nerve stimulation

Maximal compound action potential (*M*_max_) was acquired from electrical stimulation (DS7AH, Digitimer Ltd, Welwyn Garden City, UK) delivered in square pulses (400 V, 500 μs) to the right musculocutaneous nerve. Self-adhesive circular electrodes (30 mm diameter, PolarTrode, Niva Medical Ltd, Espoo, Finland) were placed over the supraclavicular fossa (cathode) and acromion (anode). Stimuli were given starting at 20 mA and increased in 10 mA increments until the right biceps brachii M-wave responses plateaued. Subsequently, 3 supra-maximal stimulations were delivered at 125% of the final intensity 5–10 s apart. *M*_max_ was reported as the largest peak-to-peak amplitude (mV) of the 3 supra-maximal stimulations. *M*_max_ area (mV·ms) was calculated from EMG onset to the last time the signal crossed baseline EMG activity (mean EMG 200 ms prior to stimulation).

### StartReact test

The StartReact test purportedly probes the reticulospinal system via a startling auditory stimulus (Tapia et al. 2022; Valls-Solé et al. 1995). The StartReact test was replicated from a previously tested protocol (Baker and Perez 2017), where three test cues were used: light only (V), light with a quiet sound (VA; 80 dB, 500 Hz, 50 ms), and light with a loud sound (VS; 120 dB, 500 Hz, 50 ms). Sounds were delivered from a speaker (JBL IRX112BT active 12″ speaker, Northridge, USA), and 8 white LED lights in a diamond formation (3.5 × 3 cm) were positioned at eye-level 1 m in front of the participant. The sound took 3.7 ms to arrive to the subject owing to the delay within the pc-speaker and the speed of sound. Participants were instructed to flex at the elbow “as fast as possible” in response to the light cue. Two to three familiarization trials were conducted for each cue prior to commencing the test. During the test, twenty trials of each cue were presented in a pseudorandomized order with an inter-stimulus interval (ISI) of ~ 8 s, giving the same order to each participant. Previous iterations of the StartReact test have used the sternocleidomastoid muscle as an indicator of startle reflex activation. However, this method is often unreliable (Baker and Perez 2017; Choudhury et al. 2019). The StartReact effect is subtly different from the overt startle reflex: it does not habituate with repeated presentation (Valldeoriola et al. 1998) and is not affected by pre-pulse inhibition (Valls-Solé et al. 2005). We did not, therefore, use sternocleidomastoid recordings in this study.

Normalized changes in the gain of reticulospinal outputs were reported as a ratio of reaction times for the test cues ([V–VS]/[V–VA]) (Baker and Perez 2017; Fisher et al. 2013) defined in this study as RST gain. EMG data were analyzed semi-automatically using custom MATLAB scripts (R2023b, MathWorks Inc, Wisconsin, USA). The script applied a 500 Hz low-pass filter to the unrectified EMG signal. Reaction time was identified as the onset of rectified EMG activity exceeding 7 standard deviations (SDs) above the mean 200 ms before the stimulus. Each trial was examined visually, while blinded to the cue type, to confirm automatic placement. The rate of force development (N·s^−1^) was calculated over three time periods (0–50, 50–100, and 0–100 ms) and converted to a rate of torque development (RTD) based on each individual’s lever arm distance (N·m·s^−1^). EMG root-mean-square (EMGrms) amplitudes and mean power frequency (MPF) were also analyzed over the same time-windows (0–50, 50–100, and 0–100 ms) following EMG onset.

### Transcranial magnetic stimulation

Single-pulse TMS paradigms were used to investigate motor responses in the biceps brachii muscle. Participants performed isometric elbow flexion at 10% of MVC during all TMS protocols by following visual force feedback on a monitor 1 m in front at eye level. Stimuli were delivered using a figure-of-eight coil (70 cm winding diameter, D70 Alpha Flat coil) and Magstim BiStim^2^ magnetic stimulator (Magstim Co Ltd, Whitland, UK) operating in single-pulse mode. Monophasic current waveforms stimulated the left motor cortex. The coil was initially oriented 45° to the parasagittal plane, inducing a PA current. Coil and head placement were tracked using a Polaris Vicra camera (Northern Digital Inc, Waterloo, Canada) and Localite TMS Navigator system (version 3.3.32, Localite GmbH, Bonn, Germany). Sets of three motion capture markers were fixed to both a pair of glasses worn by the participant and the coil. The hotspot was defined as the location, where the largest MEPs were elicited in the right biceps brachii using a stimulator output of 40–50%. Active motor threshold (aMT) was determined as the intensity at which ≥ 5/10 consecutive stimuli elicited a sufficient MEP (> 100 μV) (Rossini et al. 2015).

The recruitment curve test involved sets of 10 TMS pulses delivered at 100%, 110%, 120%, 140%, 160%, and 180% of aMT to assess corticospinal excitability (Devanne et al. 1997; Ridding and Rothwell 1997). Single-pulse stimuli were given with an ISI of 5 s and a rest period of 30 s between sets. Area under the recruitment curve (AURC) was calculated using trapezoidal integration for low (100–120% aMT) and high stimulation intensities (140–180% aMT).

Next, loud acoustic stimulation was paired with single-pulse TMS to assess reticulospinal excitability by activating subcortical structures with a startling sound (Germann and Baker 2021). TMS pulses were delivered in the PA current direction at 120% of aMT. TMS stimuli were delivered alone (control MEP), or a loud sound was given starting 50 ms prior to TMS (conditioned MEP; 120 dB, 500 Hz, 50 ms). This intensity and interval, arriving to the participant ~ 46 ms prior to the TMS pulse, would be expected to produce suppression in MEP size (Furubayashi et al. 2000; Germann and Baker 2021). Ten trials for each condition were presented in a pseudorandomized order that was consistent for each participant.

Thereafter, following 2–3 min rest, the coil was rotated 180° while maintaining the same hotspot for AP current stimulation. AP current stimulation has been shown to evoke later I-waves, possibly as a result of enhanced cortico-reticular pathway activation compared to PA current stimulation (Cirillo and Perez 2015; Federico and Perez 2017). The AP MEP latency is 1.5 ms longer on average than from PA current. The AP aMT was acquired for the new coil position using the same criteria as for the PA current. Once the AP aMT was determined, 10 stimuli were given at 120% aMT with an ISI of 5 s. If MEP onset latency from AP current stimuli was < 0.5 ms longer than PA stimuli at 120% aMT, the participant’s PA and AP MEP latencies were excluded from further analyses (*n* = 3).

TMS data were analyzed using custom MATLAB scripts. All EMG responses were reviewed visually, and only accepted MEPs (> 100 μV) were included into subsequent analyses. Such non-identification of MEPs only affected trials at 100% aMT. MEP onset, offset, and silent period were manually identified from the mean rectified MEP of all trials for the given condition. MEP onset was marked as the point, where EMG activity exceeded 2 SDs above mean EMG activity 200 ms prior to stimulus (i.e., baseline EMG), and offset was defined as the first return to baseline EMG. For measures of MEP size (peak-to-peak amplitude and area), the median of 10 trials was normalized to *M*_max_ (MEP/*M*_max_) for each participant and used in statistical analyses. The silent period was defined as the time from stimulus onset to the return to baseline EMG. The silent period (ms) was normalized to MEP area/*M*_max_ as the preceding MEP size effects silent period duration (Orth and Rothwell 2004). The area of the first 5 ms of MEP (MEP_5ms_) in response to recruitment curve stimuli was also obtained, and the median of 10 trials was used for statistical analyses.

### Statistical analysis

All statistical analyses were conducted using SPSS Statistics (version 28.0, IBM, Armonk, NY). Normality of data was assessed using the Shapiro–Wilk test. Data that were not normally distributed were log-transformed before further statistical tests. Independent-samples *t* tests were used to examine differences in physical characteristics, RST gain, MEP sizes, and TMS with loud acoustic stimulation responses between groups. Paired-samples *t* tests were conducted for comparisons between PA vs. AP current aMT, MEP size and silent period duration. Two-way repeated-measures ANOVAs were used in StartReact analysis to examine the effect of cue (V, VA, and VS) and group on reaction time. Two-way repeated-measures ANOVAs were also used to determine the effect of TMS intensity and group on MEP responses from the recruitment curve test. Three-way repeated-measures ANOVAs were implemented to assess the effect of StartReact cue, group, and time-window (0–50, 50–100, and 0–100 ms) on RTD, EMGrms, and MPF. Huynh–Feldt correction was used when sphericity was not assumed (Mauchly’s test). When a significant *F* value for the condition was observed, Bonferroni post-hoc tests were used to determine, where the difference lay. The alpha level was set to 0.05. Hedges’ *g* was used to calculate effect sizes with the following cutoffs for interpretation: small effect < 0.2, medium effect = 0.2–0.8, and large effect > 0.8 (Hedges and Olkin 1985). All data are presented as mean ± SD unless indicated otherwise.

## Results

### Physical characteristics and maximal torque production

Physical characteristics and maximal strength parameters are shown in Table 1. Based on self-report, 8 females were tested 14–28 days (i.e., luteal phase) following the start of their last menstruation, and the remaining 3 were amenorrhoeic (2 with intrauterine devices and 1 arm implant). The groups were of similar age, height, and body mass.

**Table 1:** Physical characteristics of the strength-trained and untrained groups

	Strength-trained	Untrained	*p*
*n* (male/female)	15 (11/4)	18 (11/7)	
Age (years)	31.5 ± 5.9	31.6 ± 4.4	0.990
Height (cm)	175.9 ± 7.7	175.4 ± 9.2	0.848
Body mass (kg)	85.5 ± 16.0	77.3 ± 12.7	0.115
Total SMM (kg)	40.3 ± 8.7	33.0 ± 6.3	0.010*
Right arm SMM (kg)	4.2 ± 1.1	3.3 ± 0.8	0.013*
Body fat (%)	17.2 ± 5.9	24.2 ± 7.0	0.006**
Torque_max_ (N·m)	121.8 ± 40.4	84.1 ± 26.0	0.003**
Torque_max_/Right arm SMM (N·m·kg^−1^)	28.8 ± 3.7	25.4 ± 4.2	0.022*
RTD_max_ (N·m·s^−1^)	664.5 ± 293.4	484.4 ± 201.7	0.046*
RTD_max_/Right arm SMM (N·m·s^−1^·kg^−1^)	154.5 ± 44.2	145.2 ± 43.1	0.557

Values are mean ± SD

*SMM* skeletal muscle mass, *Torque*_*max*_ maximal torque production, *RTD*_*max*_ maximal rate of torque development over the first 100 ms of contraction

Significant differences between groups are identified by * (*p* < 0.05) and ** (*p* < 0.01)

The strength-trained group had significantly greater total SMM, greater right arm SMM, and lower percent body fat than the untrained group. As expected, the strength-trained group also had significantly greater absolute Torque_max_, Torque_max_ relative to right arm SMM, and RTD_max_ than the untrained group. RTD_max_ relative to right arm SMM was similar between groups.

### StartReact test

**Fig. 1:**
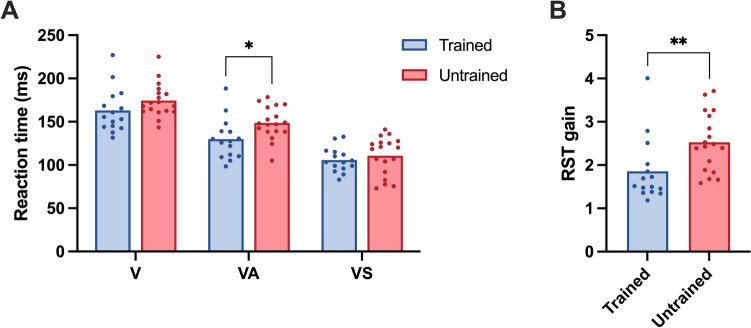
**A** StartReact reaction times under the light-only cue (V), light with quiet sound cue (VA), and light with loud sound cue (VS) in strength-trained and untrained groups. **B** Reticulospinal tract (RST) gain ([V-VS]/[V-VA]) in trained and untrained groups based on cue reaction times. Significant differences between the groups are denoted by * (*p* < 0.05) and ** (*p* < 0.01)

Two-way repeated-measures ANOVA was used to determine the effect of cue type (V, VA, and VS) and training status on reaction time. Reaction time was only affected by StartReact cue type (*F*_(1.3,41)_ = 178.0, *p* < 0.001). There was no effect of group on reaction time (*F*_(1,31)_ = 3.5, *p* = 0.073) nor interaction (*F*_(1.3,41)_ = 2.4, *p* = 0.122). Post-hoc analyses revealed significant reductions in reaction time from V to VA cue and VA to VS cue in both groups (all *p* < 0.001; trained: *g* = 2.74 and 1.49, respectively; untrained: *g* = 3.13 and 1.71, respectively; Fig. 1A). Reaction times for each cue type were as follows: V (trained: 163.0 ± 25.9 ms; untrained: 174.4 ± 19.4 ms), VA (trained: 129.8 ± 23.7 ms; untrained: 148.7 ± 18.7 ms), and VS (trained: 105.8 ± 14.0 ms; untrained: 110.6 ± 22.0 ms). Only VA reaction time was significantly shorter in the strength-trained than the untrained group (*p* = 0.016, *g* = 0.87, 95% CI [3.8, 33.9]), while V and VS were similar across groups (*p* = 0.159 and *p* = 0.465, respectively). Consequently, RST gain ([V–VS]/[V–VA]) was significantly greater in the untrained group (*t*_(31)_ = 3.2, *p* = 0.003, *g* = 1.09, 95% CI [0.05, 0.24]; Fig. 1B). These data indicate that the quiet sound cue had a more pronounced effect on reaction time in strength athletes, while the VS cue restored parity between the groups.

**Fig. 2:**
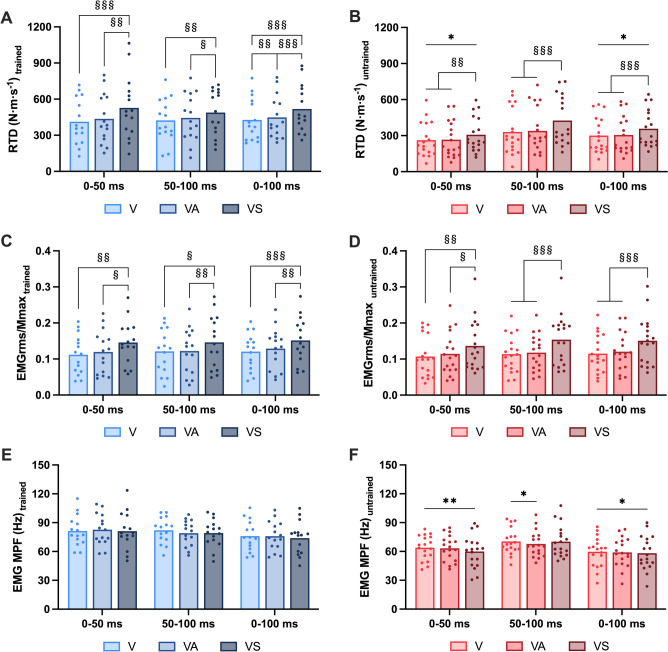
Force production and muscle activity of StartReact responses in strength-trained (**A**, **C**, **E**) and untrained (**B**, **D**, **F**) groups. Rate of torque development (RTD) (**A**–**B**), EMG root-mean-square (EMGrms) normalized to Mmax (**C**–**D**), and EMG mean power frequency (MPF) (**E**–**F**) were analyzed over three time-windows (i.e., 0–50 ms, 50–100 ms, and 0–100 ms). Responses were also compared between the three StartReact cue types: light only (V), light with quiet sound (VA), and light with loud sound (VS). Significant differences between StartReact cues are denoted by § (*p* < 0.05), §§ (*p* < 0.01), and §§§ (*p* < 0.001). Between-group differences are denoted by * (*p* < 0.05) and ** (*p* < 0.01)

Three-way repeated measures ANOVAs were used to evaluate whether cue type, training group, and time-window affected RTD, EMGrms, or MPF. RTD was affected by cue type (*F*_(1.5,44)_ = 52.9, *p* < 0.001), group (*F*_(1,30)_ = 5.8, *p* = 0.023), and time-window (*F*_(1.1,32)_ = 4.2, *p* = 0.046). However, significant interactions were not observed. Post-hoc tests revealed significantly greater RTD in response to the VS cue compared to V and VA cues for both groups across all time-windows (trained: *p* < 0.019 for all time-windows, *g* = 0.55–1.62; untrained: *p* < 0.006 for all time-windows, *g* = 0.71–1.38; Figs. 2A, B). Only cue type had a significant effect on normalized EMGrms (*F*_(1.4,43)_ = 43.4, *p* < 0.001). Similar to RTD, EMGrms was significantly greater in response to the VS cue compared to V and VA cues for both groups across all time-windows (trained: *p* < 0.016 for all time-windows, *g* = 0.80–1.19; untrained: *p* < 0.030 for all time-windows, *g* = 0.62–1.30; Fig. 2C, D).

MPF, on the other hand, was not affected by cue type. There were significant effects for group (*F*_(1,31)_ = 10.2, *p* = 0.003) and time-window (*F*_(1.4,44)_ = 11.2, *p* < 0.001), and a group × time-window interaction (*F*_(1.4,44)_ = 3.7, *p* = 0.047). MPF was significantly greater in the strength-trained than the untrained group across all time-windows and cue types (V: *p* = 0.001–0.018, *g* = 0.85–1.19; VA: *p* < 0.021, *g* = 0.83–1.28; VS: *p* = 0.002–0.014, *g* = 0.89–1.15), with the exception of the VS cue from 50 to 100 ms (*p* = 0.110; Fig. 2E, F). Overall, the VS cue enhanced RTD and EMGrms in both groups, while MPF was augmented in all conditions in strength athletes.

### Recruitment curve

Two-way repeated-measures ANOVAs were used to determine the effect of TMS intensity on MEP size and silent period. There was a significant effect of stimulation intensity for MEP peak-to-peak amplitude (*F*_(1.7,53)_ = 152.0, *p* < 0.001) and area (F_(1.8,53)_ = 127.0, *p* < 0.001). Post-hoc tests yielded significant increases in normalized MEP peak-to-peak amplitude and area (MEP/*M*_max_) across all intensities for both strength-trained (all *p* ≤ 0.001) and untrained groups (all *p* < 0.012), except for peak-to-peak amplitude between 160% (0.59 ± 0.30) and 180% aMT (0.65 ± 0.33, *p* = 0.185) in the untrained group.

**Fig. 3:**
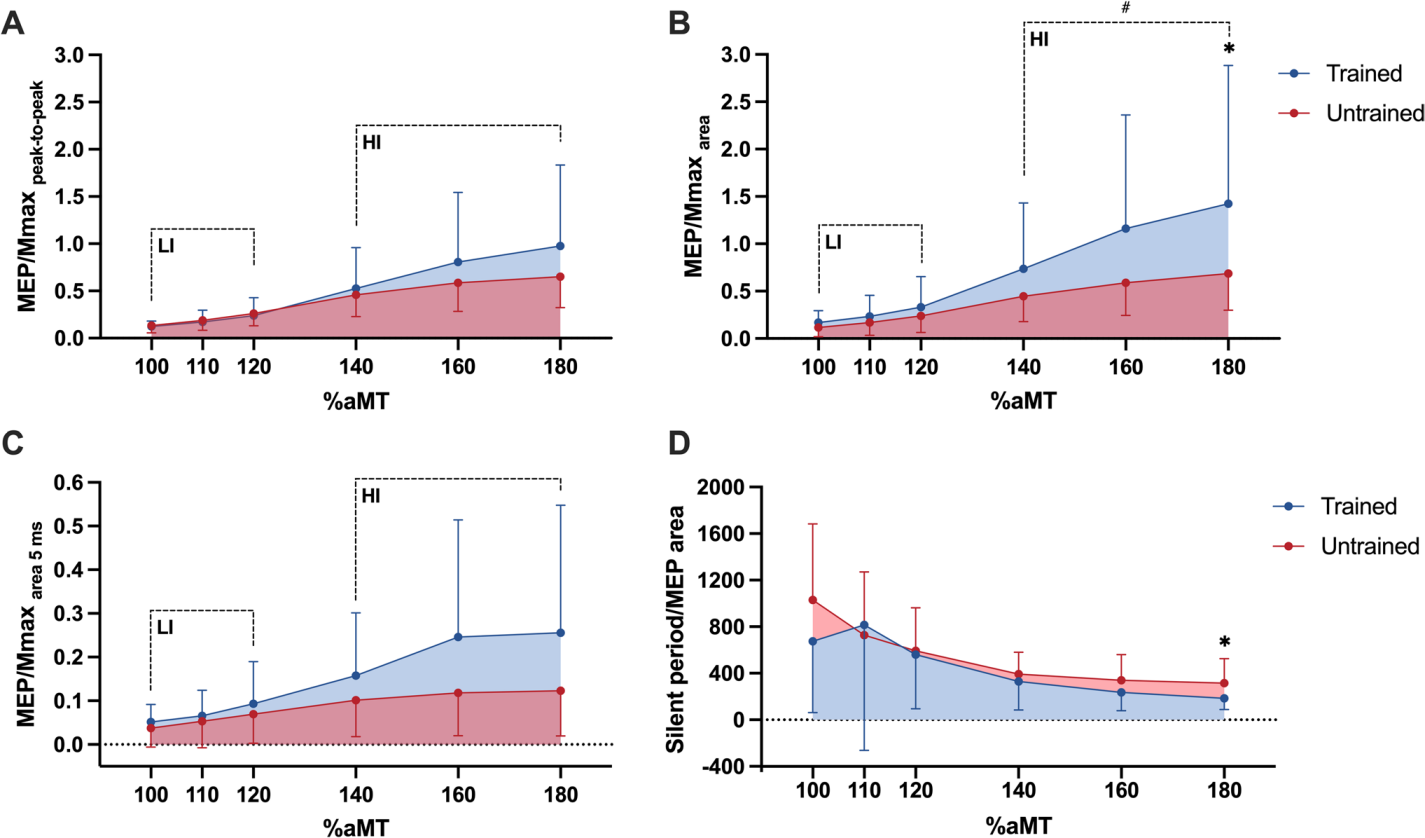
Mean motor evoked potential (MEP) size and silent period duration responses during recruitment curve tests in strength-trained and untrained groups. MEP peak-to-peak amplitude (**A**), total MEP area (**B**), and MEP area over the first 5 ms after onset (**C**) are presented normalized to Mmax. Silent period durations (**D**) were normalized to MEP area at each respective stimulation intensity. Area under the recruitment curve at low-intensity (LI: 100–120% aMT) and high-intensity (HI: 140–180% aMT) stimulations were compared between groups – with intensity ranges based on active motor threshold (aMT%). Standard deviation is represented by error bars. Significant differences between groups at a given stimulation intensity are denoted by * (*p* < 0.05) and area under the recruitment curve by # (*p* < 0.05)

There was a significant group × stimulation intensity interaction for MEP peak-to-peak amplitude (*F*_(1.8,53)_ = 4.1, *p* = 0.026), while area did not reach statistical significance (*F*_(1.7,50)_ = 3.3, *p* = 0.053). There was no main group effect for either measure of MEP size (peak-to-peak: *F*_(1,30)_ = 0.1, *p* = 0.827; area: F_(1,30)_ = 1.9, *p* = 0.177). Post-hoc tests showed a significant between-group difference in MEP area at 180% aMT (trained: 1.43 ± 1.46, untrained: 0.69 ± 0.39, *p* = 0.020, *g* = 0.86, 95% CI [0.04, 0.47]; Fig. 3A, B). In addition, there was a medium effect size at 160% aMT for MEP area (trained: 1.16 ± 1.20, untrained: 0.59 ± 0.35, *p* = 0.059, *g* = 0.67, 95% CI [−0.01, 0.44]). There was no interaction (*F*_(2.6,78)_ = 1.7, *p* = 0.186) or group effect (F_(1,30)_ = 1.4, *p* = 0.239) on normalized MEP_5ms_.

AURC comparisons showed significantly greater normalized MEP area at high-intensities (140–180% aMT) in the strength-trained (89.7 ± 90.0% aMT) than the untrained group (46.3 ± 26.4% aMT, *t*_(31)_ = 2.1, *p* = 0.048, *g* = 0.70, 95% CI [0.002, 0.42]; Fig. 3B). Conversely, there was no difference between groups in MEP_5ms_ AURC to stimuli at high-intensities (trained: 18.1 ± 19.0% aMT, untrained: 9.2 ± 7.6% aMT*, t*_(31)_ = 1.6, *p* = 0.118, *g* = 0.55; Fig. 3A, C). These recruitment curve data suggest that strength athletes had greater MEP size responses at higher stimulation intensities, but not when assessed over the first 5 ms of the MEP.

Normalized silent period was significantly affected by stimulation intensity (*F*_(1.9,53)_ = 49.3, *p* < 0.001; Fig. 3D). There was no interaction (*F*_(1.9,53)_ = 2.4, *p* = 0.104) nor effect of group (*F*_(1,28)_ = 1.7, *p* = 0.199) on normalized silent period. Notably, normalized silent period was significantly shorter at 180% aMT in the strength-trained group (183.5 ± 95.1% aMT) than the untrained group (313.9 ± 210.0% aMT, *p* = 0.035, *g* = 0.80, 95% CI [−0.45, −0.02]).

### Loud acoustic stimulation paired with TMS

**Fig. 4:**
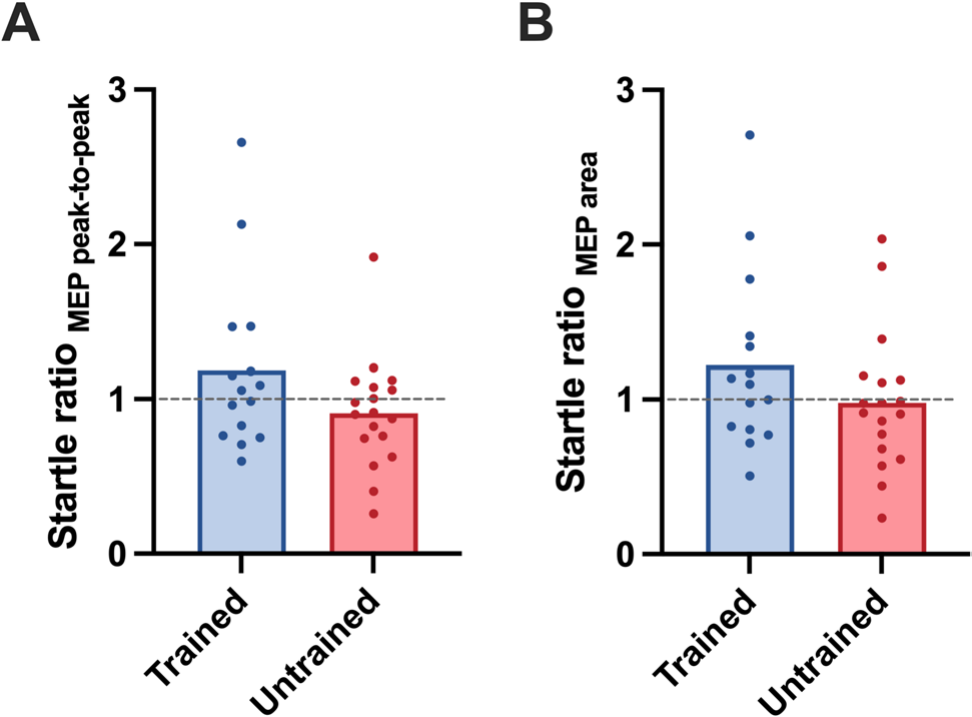
Startle ratio (conditioned MEP/control MEP) for MEP peak-to-peak amplitude (**A**) and area (**B**) in strength-trained and untrained groups. A ratio of 1.0 equates to the same MEP size from the two stimulation conditions and is indicated by the dashed line

When a loud sound preceded single-pulse TMS stimulation, 33% (5/15) of strength-trained participants exhibited > 5% MEP area suppression, while this level of suppression was observed in 50% (9/18) of the untrained group. There were no significant differences in startle-to-control ratio for MEP peak-to-peak amplitude (trained: 1.19 ± 0.56, untrained: 0.91 ± 0.36, *t*_(31)_ = 1.8, *p* = 0.088, *g* = 0.60) nor area (trained: 1.22 ± 0.58, untrained: 0.98 ± 0.45, *t*_(31)_ = 1.3, *p* = 0.164, *g* = 0.49; Fig. 4B). Thus, MEP responses to loud acoustic stimulation paired with TMS were similar between groups.

### PA and AP current orientation comparisons

Both groups had significantly greater aMTs in the AP current orientation than PA (trained: *t*_(14)_ = 11.1, *p* < 0.001, *g* = 2.70; untrained: *t*_(17)_ = 12.3, *p* < 0.001, *g* = 2.76; Table 2). There were no differences between coil orientations for normalized MEP peak-to-peak amplitude (trained: *p* = 0.290, *g* = 0.27; untrained: *p* = 0.531, *g* = 0.14), MEP area (trained: *p* = 0.107, *g* = 0.42; untrained: *p* = 0.362, *g* = 0.21), or silent period (trained: *p* = 0.846, *g* = 0.05; untrained: *p* = 0.716, *g* = 0.08) in either training group. AP-induced MEPs had significantly longer latencies than PA in both groups (trained: *t*_(13)_ = 4.5, *p* < 0.001, *g* = 1.14; untrained: *t*_(15)_ = 5.6, *p* < 0.001, *g* = 1.33). No differences were found between the strength-trained and untrained groups across MEP parameters in PA or AP orientations at 120% aMT.

**Table 2:** PA and AP current-induced MEPs at 120% aMT

	Strength-trained		Untrained	
	PA	AP	PA	AP
aMT (%)	38.7 ± 9.3	53.9 ± 12.7^§§§^	38.4 ± 7.5	54.1 ± 10.4^§§§^
MEP latency (ms)	13.8 ± 2.5	15.1 ± 2.7^§§§^	13.6 ± 2.0	14.3 ± 2.1^§§§^
MEP peak-to-peak/M_max_	0.22 ± 0.12	0.27 ± 0.18	0.28 ± 0.16	0.29 ± 0.17
MEP area/M_max_	0.32 ± 0.29	0.42 ± 0.36	0.26 ± 0.18	0.28 ± 0.18
Silent period/MEP area	495.7 ± 376.6	572.3 ± 596.3	557.6 ± 360.5	535.3 ± 361.2

Values are mean ± SD. MEP area (mV·ms) and peak-to-peak amplitude (mV) were normalized to the *M*_max_ and reported as a ratio. Silent period (ms) was normalized to MEP area and reported as a ratio

*PA* posterior–anterior, *AP* anterior–posterior, *MEP* motor evoked potential, *aMT* active motor threshold

Within-group differences between current orientations are denoted by ^§§§^ (*p* < 0.001)

## Discussion

The present study investigated corticospinal and reticulospinal excitability and whether excitability is affected by strength training status. As expected, long-term strength-trained participants had greater force production characteristics and muscle mass compared to untrained participants. The strength difference persisted when normalizing force by muscle mass, suggesting adapted neural contributions to maximum force development. MEP size differentiated between groups at higher stimulation intensities only, suggesting that polysynaptic pathways were more developed in strength-trained adults. Silent period duration, assumed to represent intracortical inhibition, was shorter in the strength-trained group at the highest stimulation intensity. Furthermore, greater response to loud acoustic stimulation in untrained adults suggests a greater reliance on the loud sound (120 dB) to activate the reticular formation. Thus, it could be argued that long-term strength-trained adults demonstrated upregulated cortico-reticulospinal functioning accompanying their enhanced force production capacity.

### Effects of loud acoustic stimulation on voluntary and involuntary responses

In the StartReact test, loud sound led to the shortest reaction times in both groups, as expected (Davis et al. 1982; Valls-Solé et al. 1995). The similar reaction times observed during the visual-only (V) cue condition indicate that voluntary, cortically mediated reaction times were comparable between groups. This supports the interpretation that group differences observed in response to VA and VS stimuli are more likely due to differential subcortical excitability, rather than baseline reaction time differences or task familiarity. While there were no differences between groups in reaction time to loud sound, the strength-trained group demonstrated shorter reaction times from the 80 dB VA cue. This led to a more pronounced reduction in reaction time from VA to VS (~ 38 vs. ~ 24 ms) in the untrained group and, consequently, a greater RST gain score. The improvements in both groups from VA to VS should be considered to represent enhanced activation of the reticulospinal tract. We interpret the data as strength-trained athletes already activating the reticulospinal tract in the absence of loud sound and the untrained adults being able to reach the level of the strength-trained group through facilitation caused by the loud acoustic stimulus.

Some work in humans has suggested that the corticospinal tract is involved in the StartReact effect (Maslovat et al. 2014; Stevenson et al. 2014), which could limit the validity of this test as a measure of pure reticulospinal function. Tapia et al. (2022) showed clearly in monkey that activity in both motor cortex and reticular formation contributed to short reaction movements, whether or not a loud sound formed part of the cue. The contribution of both tracts to any cue condition is not, therefore, in doubt. However, the loud sound affected the two centers differently, by suppressing cortical activity (shown also in human recordings, Furubayashi et al. 2000; Germann and Baker 2021) but facilitating the reticular formation. This difference allows the magnitude of the StartReact effect to measure the relative contributions of the two tracts (Tapia et al. 2022). We also acknowledge that studies have shown even non-startling stimuli can affect force production and reaction time (Jaskowski and Verleger 1993; Ulrich et al. 1998), suggesting that both cortical and subcortical systems, including potential graded activation of the reticular formation, may contribute. Furthermore, one possibility for the shorter reaction time from VA cue in strength athletes could be enhanced intersensory facilitation (Nickerson 1973) due to the VA cue. Hence, it remains inconclusive whether the data demonstrate better corticospinal or reticulospinal functioning of strength athletes.

Both groups demonstrated increased EMGrms following the VS cue compared to V and VA cues. This finding supports previous observations in biceps brachii (Walker et al. 2024) and superficial vasti (Škarabot et al. 2022) muscles during StartReact. It appears that EMG amplitude is increased through greater motor unit discharge rates (Škarabot et al. 2022). Given that the voluntary elbow flexion action in response to the cues was known to the participants, it is plausible that a preprogrammed motor response would occur. This would fit the theory of reticular formation involvement (Carlsen et al. 2004, 2009) and potentially be the mechanism behind greater discharge rates and RTD.

Mean power frequency, as a crude representation of average conduction velocity (Solomonow et al. 1990) and, thus, motor unit recruitment, demonstrated higher values in the strength-trained group in all conditions except for one time-window (VS cue over 50–100 ms). Combined with greater rates of torque development over 0–50 and 0–100 ms, this suggests that the athletes had either greater proportion of Type II motor units than untrained innately (the authors are unaware of any evidence in the literature for this) or that they could better activate higher threshold motor units during voluntary actions, as shown to occur following short-term strength training (Del Vecchio et al. 2019; Van Cutsem et al. 1998). There was no evidence that startle-inducing loud sound led to greater motor unit recruitment, although the MPF method is flawed for such interpretations over short (e.g., 50 or 100 ms) time-windows.

Conditioning TMS with loud acoustic sound is termed StartleTMS. Previously, MEP suppression has been demonstrated over 30–60 ms latencies (Furubayashi et al. 2000). However, in the same study, no suppression of transcranial electric stimulation-induced MEPs occurred over 30–60 ms latencies, which are known not to be affected by cortical excitability (Ugawa et al. 1991). Furthermore, facilitation at 80 ms was shown from transcranial electric stimulation, which is purported to be of sub-cortical origin. It may be proposed that responses during StartleTMS represent the balance between cortical suppression and reticular facilitation (Tapia et al. 2022). In the present study, no between-group differences were observed for MEP facilitation (group means = 1.19 vs. 0.91, *p* = 0.088, *g* = 0.60), and as such should be considered unresolved. Facilitated MEP responses have been shown, even from non-startling (70 dB) acoustic stimulation, when the speaker was located < 80 cm from the participant’s hand (Serino et al. 2009). Since the speaker was positioned at 1 m in the present study, it is unlikely that this is the cause of the predominantly facilitated MEP responses. Thus, it is more likely that the observed facilitated MEPs were due to a suppression: facilitation balance in favor of facilitation. While the StartleTMS test is promising, it appears insensitive and perhaps further methodological advancement is needed to optimize its usage.

### Evidence of enhanced polysynaptic excitability in strength-trained athletes

The MEP recruitment curve from PA current stimulation is considered to be dependent on axonal excitability of corticospinal neurons at lower stimulation intensities (Di Lazzaro and Rothwell 2014) and dependent on the balance of inhibitory Gamma-amino butyric acid type A (GABA_A_) receptor and facilitatory glutamate α-amino-3-hydroxy-5-methyl-4-isoxazolepropionic acid (AMPA) receptor activity at higher stimulation intensities (Boroojerdi et al. 2001; Di Lazzaro et al. 2003). There were no between-group differences in the present study for stimulation intensities < 140% aMT as assessed either by direct comparison or as an AURC comparison. Differences were only revealed at the highest stimulation intensity and when AURC analysis was performed for 140–180% aMT intensities. Previous short-term strength-training intervention studies have shown mixed findings from testing at 120% aMT, whereas some studies that have stimulated at higher intensities have identified differences over time (Gomez-Guerrero et al. 2024a; Hortobágyi et al. 2011; Mason et al. 2020). When the present study’s MEP area data was limited to the initial 5 ms, no between-group differences were observed at any stimulation intensity. This suggests that excitability of early I-waves, preferentially influenced at lower stimulation intensities, was comparable between groups. The between-group differences emerging only at higher intensities likely reflect increased excitability of late I-waves in strength-trained athletes, which may involve polysynaptic circuits including cortico-reticular projections (Cirillo and Perez 2015; Fisher et al. 2015). Such adaptations are consistent with findings from Germann et al. (2023), who linked strength-induced facilitation in MEPs to enhanced cortico-reticular connectivity. While we recognize that the late I-wave origin may be heterogeneous (Ni et al. 2011), the convergence of our findings with prior work supports the interpretation that long-term strength training is accompanied by enhanced polysynaptic excitability, plausibly mediated via subcortical pathways.

AP current stimulation evokes motor responses with a greater contribution from late I-waves compared to the PA orientation (Di Lazzaro et al. 2001), again possibly reflecting efficacy of more indirect, subcortical pathways (Cirillo and Perez 2015; Federico and Perez 2017). In the present study, AP MEP latency was ~ 1 ms later than PA current stimulation, which agrees with previous studies (Di Lazzaro and Rothwell 2014; Federico and Perez 2017). As expected, AP aMT was at a greater stimulator output than PA current stimulation in both groups but this did not lead to larger MEP responses. Germann and Baker (2021) observed facilitated MEPs from AP, but not PA, current stimulation following an intervention involving paired auditory and muscular stimulation in the biceps brachii, suggesting that the origin was subcortical. In the present study, the strength-trained group showed weak evidence of enhanced excitability from AP current stimuli as MEP size was greater compared to PA with a medium effect size (e.g., normalized MEP area =  ~ 0.32 vs. ~ 0.42, *p* = 0.107, *g* = 0.42), whereas untrained MEP size was almost identical (e.g., normalized MEP area =  ~ 0.26 vs. ~ 0.28, *p* = 0.362 g = 0.21). Given that the stimulation intensity here was 120% aMT, and that the recruitment curve only observed differences at higher stimulation intensities (i.e., > 140% aMT), it may be worth exploring whether strength-training influences excitability to higher intensity AP stimulation in future. Although, given the typically higher aMT for AP stimulation, it may not be feasible to reach 180% aMT (e.g., 12/33 participants would have exceeded the maximum stimulator output to reach 180% aMT).

Of note, late I(_3_)-waves can be generated by different neural pathways (Ni et al. 2011). Thus, high-intensity PA and low-intensity AP current stimulation may reflect different processes that ultimately generate I_3_-waves. Observing between-group differences from one test but not from another is not necessarily contradictory. This finding may reflect specific processes/pathway(s) that are enhanced from strength training.

### Evidence of decreased cortical inhibition in strength-trained athletes

The duration of the silent period following TMS, while voluntarily contracting is thought to represent intracortical inhibition (Inghilleri et al. 1993). In particular, studies have identified GABA inhibitors, and particularly GABA_B_ receptors (Siebner et al. 1998), as playing a key role. Some recent studies have questioned whether the silent period is governed exclusively by cortical mechanisms. Although the later portion of the silent period is commonly attributed to GABA_B_-ergic mediated intracortical inhibition (Inghilleri et al. 1993; Siebner et al. 1998), evidence also points to a potential contribution from spinal circuits, including prolonged motor neuron inhibition (Fuhr et al. 1991; Gomez-Guerrero et al. 2024b; Yacyshyn et al. 2016). Therefore, while our finding of a shorter silent period at high stimulation intensity in strength-trained athletes is consistent with reduced intracortical inhibition, we recognize that spinal mechanisms may also partially underlie this difference.

Furthermore, it is important to note that the silent period duration is influenced by the preceding MEP size (Orth and Rothwell 2004). Hence, the silent period was normalized to MEP size in the present study. This is important as there were between-group differences observed in MEP responses at higher intensities. We show that long-term strength-trained athletes have shorter normalized silent period duration at 180% aMT than untrained adults, indicating that systematic strength training leads to lower GABA/GABA_B_ activity. Silent period duration at lower stimulation intensities appears unaffected by training status, which may help to explain previous mixed findings when ~ 120% aMT stimulation is typically used. Similarly, the silent period duration was unaffected by current orientation at 120% aMT. Future studies should consider whether silent period duration following AP-induced MEPs would differ between training groups at higher intensities as observed in PA stimulation, and what neurophysiological information this may provide.

Reduced silent period duration in strength-trained athletes agrees with a meta-analysis that addressed corticospinal adaptations after short-term strength-training interventions (Kidgell et al. 2017). Combining these findings supports our claim that strength training modifies polysynaptic functioning, since the earliest I-wave (I_1_-wave) is not affected by GABA activity, as reviewed by Di Lazzaro and Rothwell (2014). In our previous study (Akalu et al. 2024) comparing long-term strength-trained vs. untrained adults, a clear between-group difference was observed in the response to paired-pulse stimulation. Reduced inhibition and greater facilitation suggest modulated GABA and glutamatergic activity in strength athletes, and such evidence is complimented by the present study, where different measures and participants were used.

### Study limitations

It is likely that the methods used in the present study were not optimal to determine cortico-reticulospinal functioning. Here, we used a broad test battery of purported tests assessing reticulospinal tract excitability. However, there is no consensus on the test parameters (e.g., stimulation intensity, conditioned stimulus latency, etc.) capable to distinguish between groups, since very few data are published in humans. Consequently, the present study provides evidence for future researchers to modify the test protocol. For example, given that higher stimulation intensities demonstrated differences between groups in MEP size, evaluating AP responses at a higher intensity than at 120% aMT may be a methodological advancement.

Despite the broad test battery employed, the present study did not include tests for ipsilateral MEPs. As a portion of the reticulospinal tract does not decussate, iMEPs have been proposed to represent reticulospinal excitability and can be probed by bilateral actions (Hu et al. 2024; Maitland and Baker 2021). All electrophysiological tests performed in the present study were unilateral, isometric actions at a low force level (10% of MVC). Previously, grip strength has been related to iMEP size (Maitland and Baker 2021) and it could be suggested that this test may have been a sensitive measure to detect between-group differences in the present study, given the divergent strength levels here. Thus, inclusion of iMEP examination in future strength training studies could be encouraged.

## Conclusions

Reticulospinal gain, assessed via StartReact, showed that untrained adults benefitted more from loud acoustic stimulation compared to long-term strength-trained athletes. Shorter reaction times to the 80 dB cue among strength-trained athletes could indicate a more excitable cortico-reticulospinal system, primed for activation even without the loud acoustic stimulation. This finding was coupled with between-group differences at higher stimulation intensities (i.e., > 140% aMT), suggesting a progressive and superior polysynaptic contribution to MEP size in the strength-trained group. Combined, it appears that long-term strength training is characterized by greater cortico-reticulospinal excitability/functioning. Finally, some, albeit limited, evidence (silent period duration at 180% aMT) suggests that strength-trained adults may have had lower cortical inhibition than untrained adults.

## Data Availability

Data is available upon reasonable request from the corresponding author.

## Abbreviations

aMT: Active motor threshold
AP: Anterior–posterior
AURC: Area under the recruitment curve
CST: Corticospinal tract
EMG: Electromyography
EMGrms: EMG root-mean-square
ISI: Inter-stimulus interval
MEP: Motor evoked potential
*M*_max_: Maximal compound action potential
MVC: Maximal voluntary contraction
PA: Posterior–anterior
RST: Reticulospinal tract
RTD: Rate of torque development
TMS: Transcranial magnetic stimulation
V: Visual
VA: Visual–auditory
VS: Visual–startle
